# Supplementary material for: Proline Protects Boar Sperm against Oxidative Stress through Proline Dehydrogenase-Mediated Metabolism and the Amine Structure of Pyrrolidine
Source: Animals (Basel). 2020 Sep 1;10(9):1549. doi: 10.3390/ani10091549 (PMC7552335; doi:10.3390/ani10091549)
Supplement: Supplementary file 1 [file animals-10-01549-s001.zip › supplementary information.docx]

**Supplementary materials**

**Table 1S** Effect of proline on the total motility of boar sperm during liquid storage

| Proline (mM) | D1 | D3 | D5 | D7 | D9 |
| --- | --- | --- | --- | --- | --- |
| 0 | 88.08±0.53^b^ | 83.85±0.77^c^ | 75.80±0.70^c^ | 67.18±0.86^d^ | 60.67±1.98^c^ |
| 25 | 90.25±0.39^a^ | 87.26±0.80^b^ | 81.67±1.88^b^ | 74.41±0.29^c^ | 66. 73±0.86^ab^ |
| 50 | 90.33±0.63^a^ | 88.82±0.55^a^ | 85.36±1.32^a^ | 78.58±0.74^b^ | 69.60±0.68^a^ |
| 75 | 90.14±0.31^a^ | 89.21±0.77^a^ | 86.19±1.96^a^ | 81.31±1.55^a^ | 72.26±0.74^a^ |
| 100 | 89.69±0.55^a^ | 87.49±0.53^ab^ | 83.16±1.94^b^ | 77.34±1.67^b^ | 68.12±0.74^ab^ |
| 125 | 88.55±0.82^b^ | 85.68±0.74^c^ | 78.38±0.63^c^ | 70.46±2.52^d^ | 60.82±0.32^c^ |

Samples were supplemented with different concentrations of proline and stored at 17 °C for 9 days. D1 means detection at the first day of storage, others by that analogy. Values are expressed as mean ± SEM of three independent experiments. Different superscript letters in same column denote significant differences in statistics (*P* < 0.05).

**Table 2S** Effect of proline on the progressive motility of boar sperm during liquid storage

| Proline (mM) | D1 | D3 | D5 | D7 | D9 |
| --- | --- | --- | --- | --- | --- |
| 0 | 73.53±0.85^c^ | 70.47±0.93^c^ | 62.59±0.94^d^ | 57.08±0.58^b^ | 51.29±1.47^c^ |
| 25 | 76.38±0.94^b^ | 73.93±0.56^b^ | 67.66±1.05^c^ | 58.83±1.49^b^ | 53.09±0.75^bc^ |
| 50 | 78.77±0.39^a^ | 75.64±0.15^a^ | 70.80±0.78^b^ | 62.72±2.17^a^ | 56.04±1.41^b^ |
| 75 | 79.38±0.68^a^ | 77.59±0.80^a^ | 73.47±0.50^a^ | 69.96±1.54^a^ | 59.04±2.19^a^ |
| 100 | 76.28±0.82^b^ | 73.20±0.99^b^ | 67.88±0.56^c^ | 61.96±2.22^a^ | 55.69±1.46^b^ |
| 125 | 74.82±0.58^bc^ | 70.39±0.58^c^ | 63.03±1.81^d^ | 57.22±2.39^b^ | 52.13±1.08^c^ |

Samples were supplemented with different concentrations of proline and stored at 17 °C for 9 days. D1 means detection at the first day of storage, others by that analogy. Values are expressed as mean ± SEM of three independent experiments. Different superscript letters in same column denote significant differences in statistics (*P* < 0.05).

**Table 3S** Effect of proline on the motility parameters of boar sperm at the fifth day of storage

| Proline (mM) | VCL  (μm/s) | VSL  (μm/s) | VAP  (μm/s) | LIN  (%) | WOB  (%) | BCF  (Hz) |
| --- | --- | --- | --- | --- | --- | --- |
| 0 | 75.93±0.81^d^ | 37.41±1.11^c^ | 33.73±1.54^c^ | 46.16±0. 85^d^ | 45.15±2.19^bc^ | 5.82±0.37^b^ |
| 25 | 79.73±1.02^c^ | 43.70±3,71^b^ | 39.02±1.74^b^ | 53.43±0.57^c^ | 48.94±2.26^ab^ | 6.97±0.13^ab^ |
| 50 | 82.10±1.69^b^ | 46.01±2.95^b^ | 40.47±0.52^b^ | 56.37±0.29^b^ | 49.28±1.58^ab^ | 7.40±1.21^ab^ |
| 75 | 86.64±1.02^a^ | 50.25±2.10^a^ | 45.64±1.71^a^ | 59.14±0.65^a^ | 52.70±2.68^a^ | 7.74±1.21^a^ |
| 100 | 79.59±0.26^c^ | 45.31±2.69^b^ | 39.35±1.74^b^ | 56.52±0.24^b^ | 50.42±1.30^a^ | 6.76±0.37^ab^ |
| 125 | 75.00±0.04^d^ | 37.95±2.74^c^ | 32.96±0.54^c^ | 45.95±0.88^d^ | 43.94±0.74^c^ | 6.36±0.11^ab^ |

Samples were supplemented with different concentrations of proline and detected at the fifth day of storage at 17 °C in vitro. Values are expressed as mean ± SEM of three independent experiments. Different superscript letters in same column denote significant differences in statistics (*P* < 0.05).

**Supplementary figures**


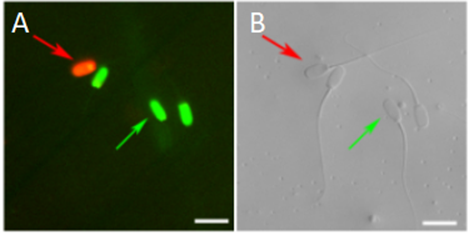


**Fig. 1S.** Photomicrographs of sperm stained with PI and SYBR-14. (A) Merged photograph of sperm stained with PI and SYBR-14. (B) Bright field image. Sperm with intact membrane (noted with green arrow) was stained with SYBR-14 only while sperm with damaged membrane (noted with red arrow) was stained with PI. Scale bars represent 20 µm.


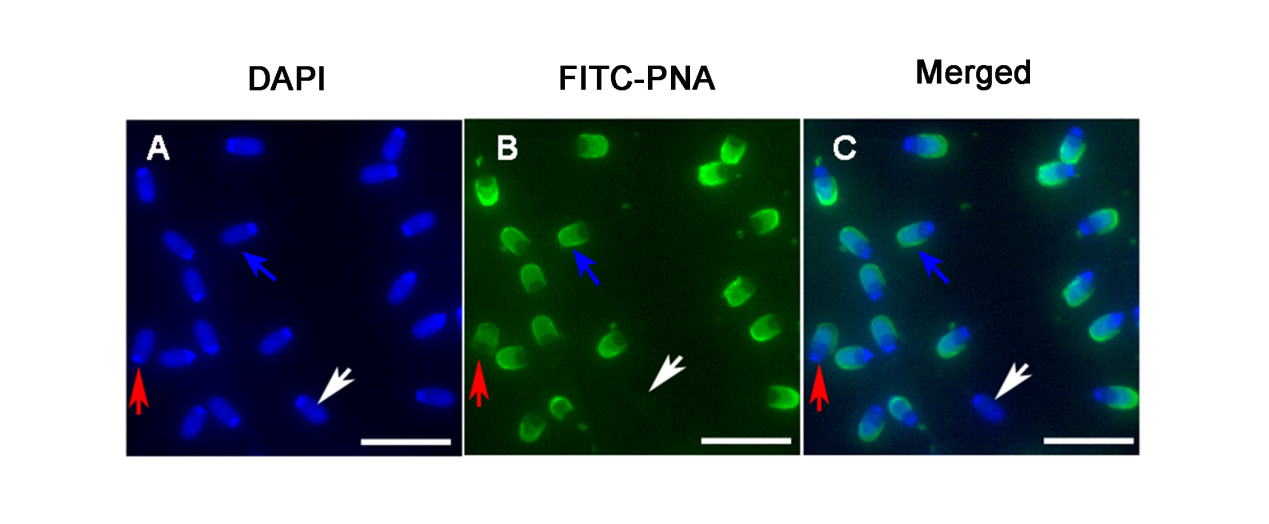


**Fig. 2S** Photomicrographs of the sperm stained with DAPI and FITC-PNA. (A) Photograph of sperm stained with DAPI. (B) Photograph of sperm stained with FITC-PNA. (C) Merged photograph. Sperm with intact acrosome were indicated by blue arrow in these pictures while sperm with damaged acrosome were indicated by white arrow. And sperm noted with red arrow were partially damaged on acrosome. Scale bars represent 20 µm.


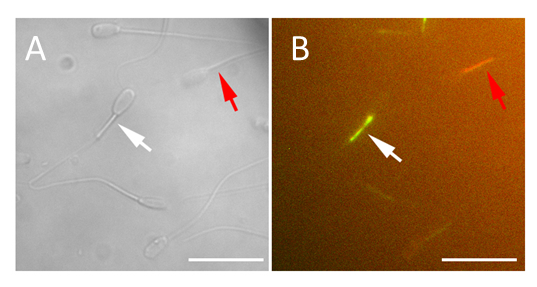


**Fig. 3S** Photomicrographs of the sperm stained with JC-1. (A) Bright field image. (B) Fluorescent image of sperm stained with JC-1. Red arrow noted sperm was high in MMP levels while white arrow noted one was low. Scale bars represent 20 µm.


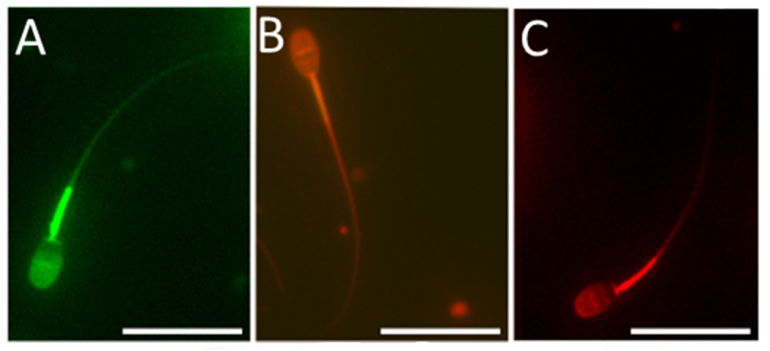


**Fig. 4S** Photomicrographs of the sperm stained with BODIPY 581/591 C11. (A) Seriously oxidized sperm. (B) Partially oxidized sperm. (C) Unoxidized sperm. Scale bars represent 20 µm.


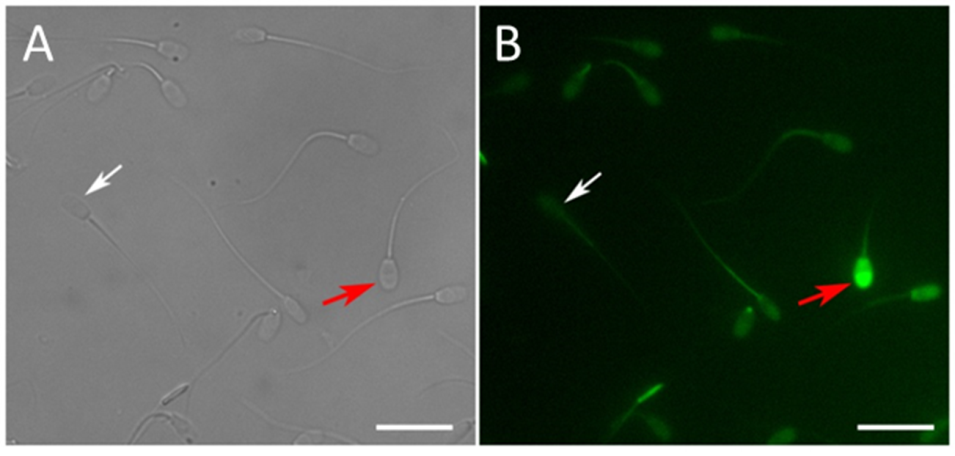


**Fig. 5S** Photomicrographs of the sperm stained with DCFH-DA. (A) Bright field image. (B) Fluorescent image of sperm stained with DCFH-DA. Sperm noted with red arrow was much higher in ROS levels than that noted with white one. Scale bars represent 20 µm.


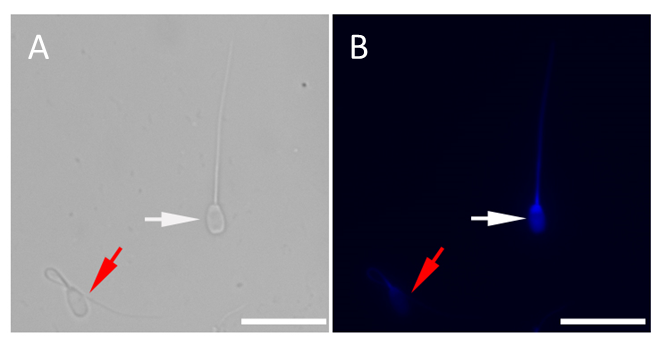


**Fig. 6S** Photomicrographs of the sperm stained with DNTB. (A) Bright field image. (B) Fluorescent image of sperm stained with DNTB. Sperm noted with white arrow was much higher on GSH levels than that noted with red one. Scale bars represent 20 µm.
